# Interdisciplinary assessment and management of a patient with a fibrous gingival enlargement of unknown origin: A case report

**DOI:** 10.1002/ccr3.2605

**Published:** 2019-12-12

**Authors:** Anna Greta Barbe, Gabriele Röhrig, Lena Hieggelke, Michael Johannes Noack, Sonja Henny Maria Derman

**Affiliations:** ^1^ Department of Operative Dentistry and Periodontology Center of Dental Medicine University of Cologne Köln Germany; ^2^ Geriatric Diagnostic Center MVZ Medicum Köln Ost Cologne Germany; ^3^ Institute of Pathology University Hospital Cologne Cologne Germany

**Keywords:** alpha thalassemia, folic acid, gingival enlargement, plasma cell gingivitis, sickle cell anemia

## Abstract

A gingival enlargement of unclear cause could only be diagnosed after interdisciplinary cooperation as plasma cell gingivitis of unknown origin. Interdisciplinary approaches remain crucial when diagnosing rare gum diseases.

## Graphical abstract

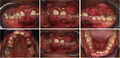

## 1 INTRODUCTION

This case report of a patient presenting with an idiopathic fibrous gingival enlargement (GE) describes the demanding periodontal diagnostic approach required to identify and treat relevant risk factors and the subsequent surgical therapy. It also presents additional knowledge gained and recommendations regarding organizational structures.

## 2 CASE REPORT

### 2.1 Clinical presentation

**Figure 1:**
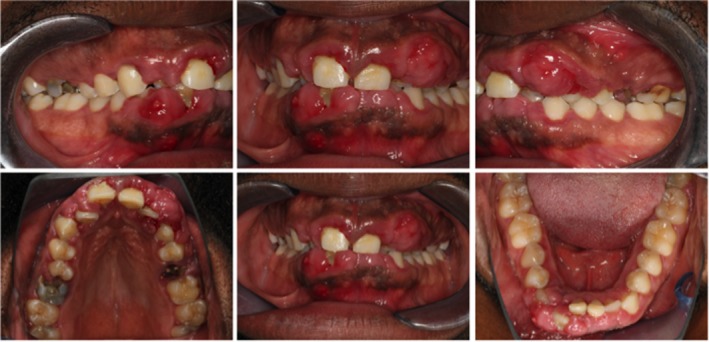
Initial presentation before treatment of a patient with deep bite and anterior crowding, multiple carious lesions, with the main concern of a massive fibrous enlargement that had been present for five years. Epuloid‐like enlargements with signs of inflammation and eroded gingiva occurred in regions 22/23 and 42/43

**Figure 2:**
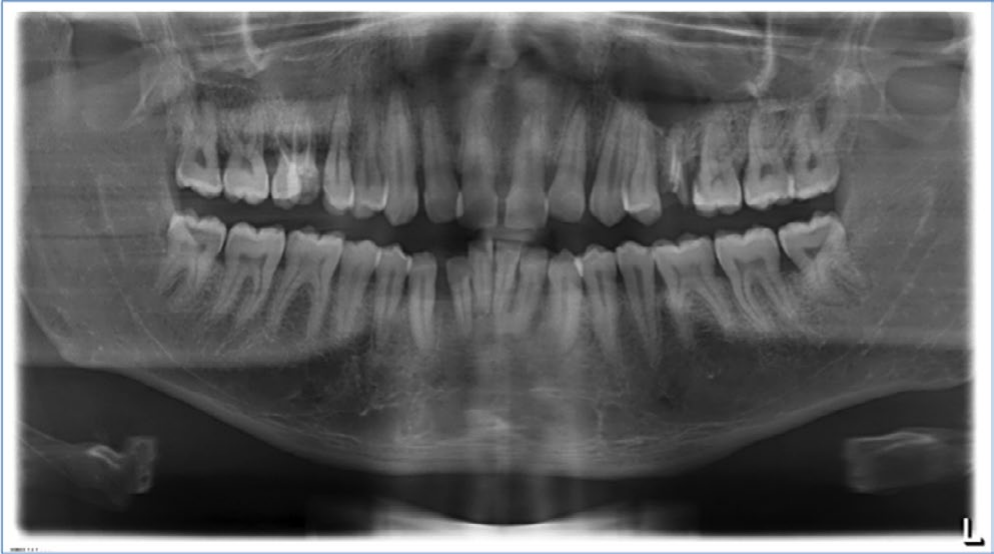
Orthopantomogram of the patient with diverse carious lesions with conservative treatment needs, and root remnants 16 and 25 with radicular cysts

In November 2018 at the University Hospital Cologne, a 33‐year‐old man from Mozambique presented with a massive, predominant, localized fibrous GE with signs of inflammation that had existed for around five years. The patient had insufficient oral hygiene (Figure 1), but no other obvious relevant risk factors (ie, no known pre‐existing conditions, no medical history, no prescribed medication intake, no family or orthodontic treatment history). An oral examination was performed, including periodontal status, preparation of X‐ray images (Figure 2) and creation of a jaw model to produce wound dressing and prosthetic planning (Figure S1). In addition to necessary treatment of carious lesions, extraction of root remnants (16 and 25 in radicular cysts), and planned periodontal therapy with gingivectomy followed by wound dressing, the patient presented to the internist and hematologist to rule out underlying hematologic disease and HIV infection as a suspected cause of hyperplasia.

### 2.2 Case management

**Figure 3:**
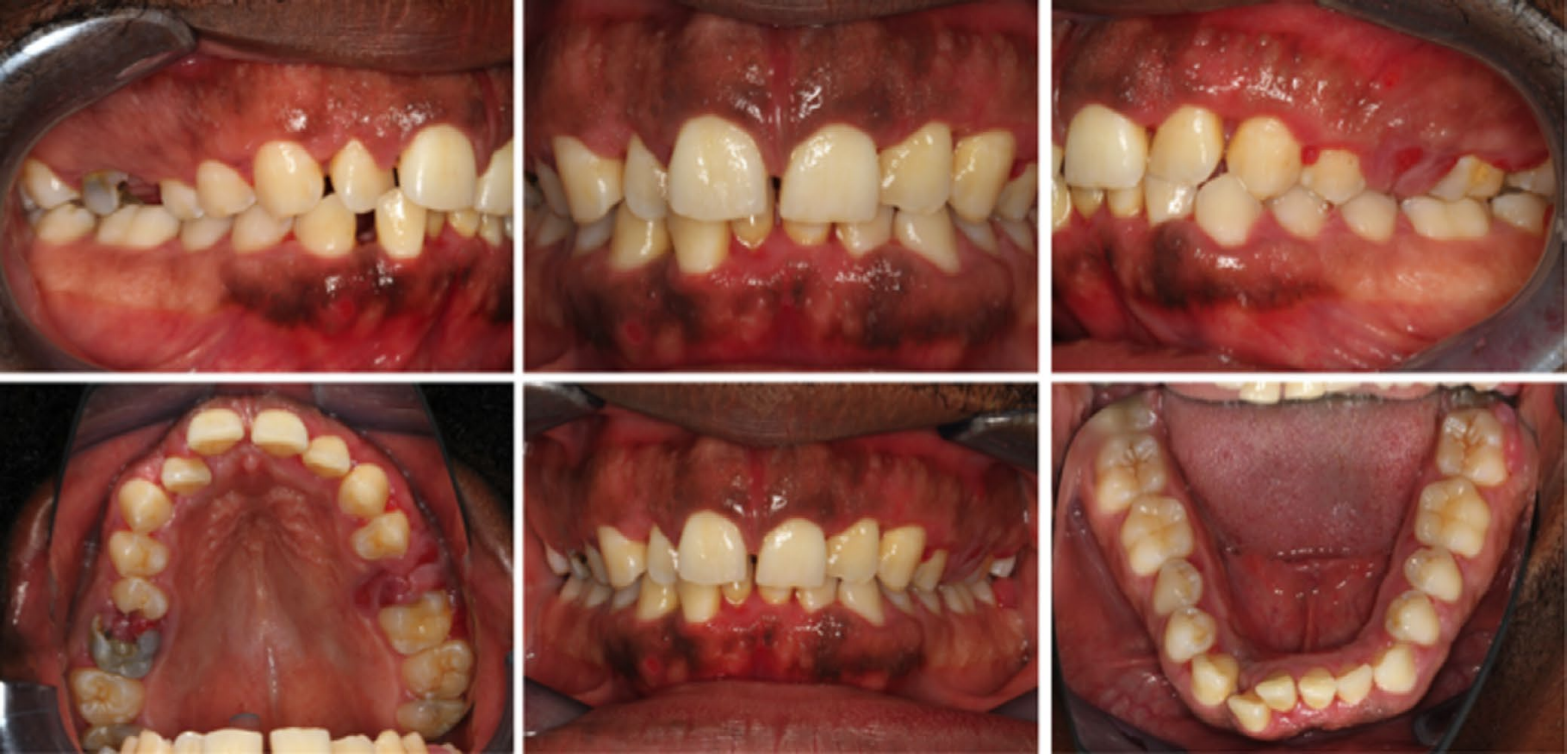
Presentation of the patient one month after gingivectomy. Since the patient decided to have the root remnants removed and therapy of the cysts of the upper jaw after gingivectomy, gingivectomy at these special sites were left out so it could be performed at the same appointment

**Figure 4:**
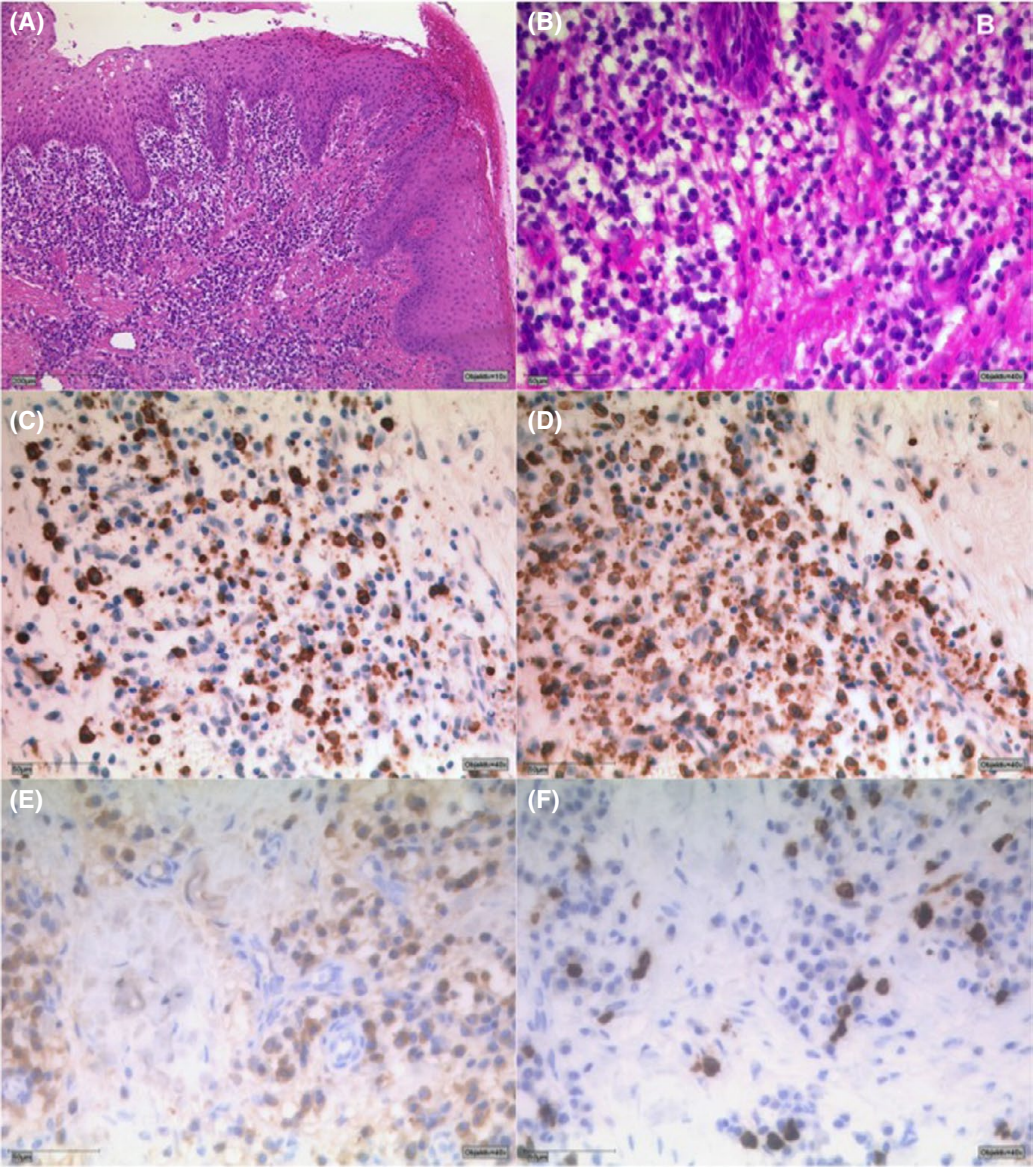
Superficial erosion of the squamous epithelium with dense plasma cell‐rich inflammatory cell infiltrate of the fibrous stromal connective tissue (H&E x 25) (A), (H&E x 400) (B). Immunohistochemistry for kappa chain (C), for lamba chain (D), for IgG (E) and IgG4 (F)

Between November 2018 and February 2019, periodontal therapy was initiated including extensive oral hygiene instructions and professional tooth cleaning every four weeks. Treatment of carious lesions and root extractions were carried out at the request of the patient at the family dentist. Once a clean oral hygiene situation had been achieved (February 2019), gingivectomy of the upper jaw was performed followed by gingivectomy of the lower jaw (March 2019), with no complications (Figure 3). The excised gingiva was coarse, felt like heavily scarred tissue when cut, and was hard to remove. The excised gingiva was sent for histopathological examination. The histopathological examination of the excised gingiva lesion (hematoxylin and eosin stain; Figure 4) revealed a stratified acanthotic squamous epithelium with slight parakeratosis and focal erosion. The underlying stromal connective tissue showed collagen‐rich fibrosis and a superficial accentuated infiltrate of inflammatory cells, predominately plasma cells accompanied by a few lymphocytes. Overall, neutrophils were absent except for the small area of surface erosion, which contained some intraepithelial neutrophils. Eosinophils were not detected. Immunostaining for kappa and lambda light chains revealed a polyclonal plasma cell population. Immunoglobulin (Ig) G and IgG4 immunostaining supplemented the analysis. Immunoglobulin IgG is elevated in patients with sickle cell disease and may help discern a sickle cell crisis from stable disease. For exclusion of IgG4 associated autoimmune disease, IgG subtype has also been analyzed. The IgG4/IgG ratio value was a maximum of 0.279 in one HPF, considering 10 different HPFs. The GE was diagnosed as plasma cell gingivitis due to the histologic findings and the exclusion of other differential diagnoses.

During anti‐infective therapy, the patient visited the internist and hematologist in January 2019. The family dentist was also contacted regarding regions 16 and 26 for planning of root extraction in this area. Laboratory results (Table 1) revealed a microcytic (low MCV), hypochromic (low MCH) erythrocytosis without anemia (normal hemoglobin (Hb) values), as well as deficiencies of vitamin D (calcifediol) and folic acid. The patient received folate and vitamin D supplementation. An HIV infection was excluded. Physical examination was unremarkable, and no clinical symptoms were reported. The red blood test results encouraged further blood analysis by the hematologist, including Hb electrophoreses and parameters of iron status. Iron deficiency was excluded, but Hb electrophoresis revealed that the patient was a heterozygous sickle cell hemoglobin (HbS) carrier, and a heterozygous alpha thalassemia minima were detected.

**Table 1:** Pathologic laboratory parameters, including electrophoresis results for hemoglobin A1 and A2 and molecular genetic alpha chain analysis for genotype assessment

Parameter	Test result	Interpretation	Reference values
Hemoglobin (g/dL)	14.7	Normal	13.5‐17.5
MCV (fL)	74	Low	80‐96
MCH (pg)	23	Low	28‐33
Calcifediol (ng/mL)	84	Low	>20 L
Folic acid (ng/mL)	3.5	Low	>5
Hemoglobin A2 (%)	3.6	Elevated	1‐3.2
Hemoglobin F (%)	0.2	Normal	<0.5
Alpha‐globin chain genotype	‐a/aa	a^+^ Thalassemia minima (aa/aa)	

Abbreviations: MCH, mean corpuscular hemoglobin; MCV, mean corpuscular volume.

The patient did not present to the family dentist for further care of the carious lesions, as originally requested, but only for the extraction of the root remnants 16 and 26 (February 2019). Therefore, the restorative therapy was performed at the dental clinic (May 2019; Figure S2). At the request of the patient, a visit to the orthodontics department of the University Hospital took place before the remaining prosthetic planning for the treatment of regions 16 and 26. Here, Invisalign^®^ therapy for optimizing the upper front jaw combined with close recall intervals for maintaining optimal oral hygiene and care was planned and initiated.

### 2.3 Clinical outcomes

The long‐term prognosis of this case remains unclear. At six months after gingivectomy, the treatment outcome had been maintained with no signs of recurrence. We assume that inadequate biofilm control by the patient—a main etiologic factor for GE—will contribute to an increased risk of recurrence. To rule out this risk and improve the prognosis, the patient attends a maintenance therapy session every three months, including dental prophylaxis. He is also seeking orthodontic treatment because of his increased esthetic awareness. This therapy will reduce the local factor of tooth crowding and improve the possibility of proper biofilm control. In addition, as folic acid and vitamin D deficiency may be one of the underlying factors for the development of GE, supplementation may help to stabilize long‐term treatment success. However, time will reveal the long‐term outcomes and will provide information regarding the impact of the underlying hematological conditions on his gingiva.

## 3 DISCUSSION

In this case report, the intraoral diagnosis of a massive fibrous GE without an obviously identifiable cause, and the consequent involvement of relevant interdisciplinary specialists, made it possible for the patient to receive the easy‐to‐treat diagnosis of vitamin D and folate deficiency, and the important and restrictive internal‐hematological diagnosis of being a sickle cell carrier with alpha thalassemia. The patient also benefited from periodontal therapy which, in addition to significantly improving oral hygiene, promoted the awareness of oral esthetics, resulting in his request for additional orthodontic treatment. Finally, this case demonstrates a possible approach to interdisciplinary care and addresses necessary steps, as demonstrated in Table 2. Although decision trees already exist regarding differential diagnosis of gingival enlargement,^1^ an additional approach regarding the organizational structure and clarifying the responsibilities is needed. Our case report provides first evidence that when diagnosis of GE depends on several disciplines and underlying factors, a guideline regarding standardized stepwise approaches for interdisciplinary management is necessary. Thus, Table 2 gives an example of such a stepwise approach that can be easily adapted to similar situations that require interdisciplinary care.

**Table 2:** Stepwise approach for the interdisciplinary management of patients with gingival enlargement of unknown origin

	Dentist/periodontist	Internist/hematologist
Detection of gingival enlargement	X	X
Preliminary diagnosis by clinical characteristics and identified risk factors including medical history (eg, decision tree)^1^	X	
Hematologic parameters with relevant systemic findings		X
Interdisciplinary board to establish the lead discipline, define the diagnosis and develop a treatment plan^a^	X	X
Anti‐infective periodontal therapy	X	
Therapy of systemic findings^b^		X
Gingivectomy (if necessary) and histopathologic confirmation of diagnosis	X	
Risk‐based supported periodontal therapy	X	
Regular controls of systemic findings/therapy		X

a In our case, the lead discipline was the periodontist; in other cases, it may be the internist or hematologist.

b In our case, supplementation with folic acid and vitamin D.

The appropriate and successful treatment of GE depends on correctly diagnosing the cause of the enlargement.^2^ The clinical appearance of our case suggested that it could have been drug‐induced GE, associated with leukemia, or accompanied by a genetic disorder (Table 3).^1^, ^3^, ^4^ In addition, areas were found with signs of reactive lesion and epuloid character (regions 22/23 and 42/43).^3^ As GEs are various in origin and clinical appearance (Table 3), all possible diagnoses were tested for plausibility, malignancies were excluded by blood testing and histopathology, and the treatment plan was formed based on possible diagnoses. Gargiulo et al described three types of origins for plasma cell gingivitis: (a) allergenic, (b) neoplastic, and (c) of unknown origin.^5^ As allergenic and neoplastic origins were excluded by blood tests and histopathologic findings, the diagnosis of “plasma cell gingivitis of unknown origin” remained. It is important to recognize that it is often hard to identify a single underlying factor, since there may be many, and relevance and causality are not easy to determine.

**Table 3:** Allocation of the classification of regional/generalized gingival enlargements (GE) by Agrawal^1^ to the recent Classification of Periodontal and Peri‐Implant Diseases and Conditions 2017

Regional or generalized gingival enlargements^1^		Classification of periodontal and peri‐implant diseases and conditions 2017^3^	
Inflammatory GE		Gingivitis, dental plaque induced	Associated with biofilm alone (2A)
GE in mouth breathers			Mediated by local risk factors (2Bii)
Fibrotic GE	Drug‐induced GE		Drug influenced GE (2C)
	Genetic disorders associated with GE—hereditary gingival fibromatosis	Gingival diseases—nondental plaque induced, genetic disorders Hereditary gingival fibromatosis (3Ai)	
Conditioned GE	Hormonal	Gingivitis—dental plaque induced, mediated by systemic risk factors, sex steroid hormones (2Bi e)	
	Vitamin C deficiency	Gingival diseases, nondental plaque induced	Endocrine, nutritional & metabolic diseases, Vitamin C deficiency (scurvy) (3Fi a)
	Plasma cell gingivitis		Inflammatory and immune conditions, hypersensitivity reactions, Plasma cell gingivitis (3Ci b)
GE associated with systemic diseases	Leukemia		Neoplasms, Malignancy, Leukemic cell infiltration and Lymphoma (3Eii b + c)
	Wegeners's Granulomatosis Crohn's disease Sarcoidosis		Inflammatory and immune conditions, Granulomatous inflammatory lesions (3Ciii) Crohn's disease (3Ciii a) Sarcoidosis (3Ciii b)
	Tuberculous GE		Specific infections, Bacterial origin, Mycobacterium tuberculosis (3Bi c)

The mediating local factors for inflammatory GE (tooth crowding and the poor oral homecare before treatment) do not fully explain the massive GE in our patient. Certainly, this case cannot conclude the causality of being a sickle cell carrier with alpha thalassemia for the presence of GE, but there are some indications that there may be an association. For both hematological diagnoses, GE is reported in single cases.^6^, ^7^ In our case, it is possible that these could be modifying factors with unknown pathomechanisms and may be a source for a higher risk of recurrence. Another possible modifying factor is the folic acid deficiency,in the presence of oral biofilm, this may exacerbate gingival inflammation and thereby promote GE.^8^, ^9^ Folic acid supplements have been shown to reduce the recurrence of gingival overgrowth in phenytoin‐induced GE following gingivectomy.^9^, ^10^ Our patient received vitamin D and folic acid supplements to address his deficiency, which could potentially reduce the risk of recurrence.

The finding that our patient was a sickle cell carrier with alpha thalassemia is of clinical relevance, as he was planning to have child with his wife. Thalassemia and sickle cell disease (SCD) are endemically prevalent in tropical parts of Africa and Middle East, with an increasing number of patients in northern Europe because of worldwide migration and there is the risk of transmitting the genetic defect to their children. Therefore, genetic counseling is obligatory.^11^ Thalassemia is a genetic disorder involving abnormal hemoglobin chains.^12^ The severity of clinical manifestation depends on the extent of the genetic defect. For African people now living outside Africa in north European regions, these hematologic aberrations often lose their selective advantage and are therefore overseen and neglected in general practice.^13^, ^14^ Given the potential risk of transmitting the gene defects to the descendants and the risk of potential rheologic crisis in asymptomatic carriers of HbS or thalassemia minima, it is important to be aware of these diseases. As asymptomatic patients will not see the hematologist, dental practices might be the only place where patients present with oral symptoms. Therefore, a dentist who sees patients of African or Asian origin with a GE should consider presentation to an internist or hematologist. Vitamin D deficiency is also typical in people of African origin living in northern Europe, because of restricted vitamin synthesis due to hyperpigmentation of the skin and weak solar radiation. Vitamin D supplementation is urgently needed in such patients to prevent musculoskeletal disorders.^15^ We assume that in addition to supplementation with folic acid, this is one factor that may contribute to a healthier and stable long‐term intraoral gingival situation. The effective interdisciplinary cooperation between dentist and hematologist has been of substantial benefit for our patient.

## 4 CONCLUSIONS

The unusual form of gingival enlargement reported here, which did not meet the criteria for recent diagnostic models, was finally diagnosed as plasma cell gingivitis of unknown origin after interdisciplinary cooperation. Incidental systemic findings of sickle cell anemia, alpha thalassemia, and vitamin D and folate deficiency may be influencing factors for the development of GE. These factors serve as an example of a stepwise approach toward interdisciplinary patient care when the underlying cause of GE are not immediately obvious. Since several factors have an impact on development of GE, the long‐term prognosis of treatment success and the general health of the patient depend on identifying and treating all potential causes. An interdisciplinary approach is necessary, and patient‐centered risk factors and other influencing factors need to be optimized to reach the best‐possible treatment results. Identification of these possible risk factors, addressing them in the treatment plan, and organizing treatment and responsibilities between different disciplines is needed to initiate a risk‐based supportive therapy and assess long‐term stability.

## CLINICAL RELEVANCE

Scientific rationale: Patients who present with gingival enlargement of unknown origin need to be thoroughly examined to identify possible influencing factors.

Principal findings: Interdisciplinary management of a fibrous gingival enlargement helped to identify underlying sickle cell anemia and alpha thalassemia and vitamin D and folate deficiency and optimize treatment.

Practical implications: Close cooperation between the dental healthcare team and other disciplines may improve treatment outcomes and help patients beyond periodontal care.

## CONFLICT OF INTEREST

None declared.

## AUTHOR CONTRIBUTIONS

All authors have contributed to the work substantially either by helping with conception of the case (Barbe AG, Röhrig G, Derman S), by contributing data (Barbe AG, Röhrig G, Hieggelke L) and writing the manuscript (Barbe AG, Noack MJ, Derman SHM) or by helping with the analysis and interpretation of the clinical data (Derman SHM, Hieggelke L, Röhrig G). All authors have been significantly involved in revising the article, and have read and approved the final version of the manuscript.
