# Supplementary material for: Interdisciplinary assessment and management of a patient with a fibrous gingival enlargement of unknown origin: A case report
Source: Clin Case Rep. 2019 Dec 12;8(1):159–65. doi: 10.1002/ccr3.2605 (PMC6982473; doi:10.1002/ccr3.2605)

**Supplement 1.** Models of the initial situation, originally used for wound dressing and orthodontic planning.


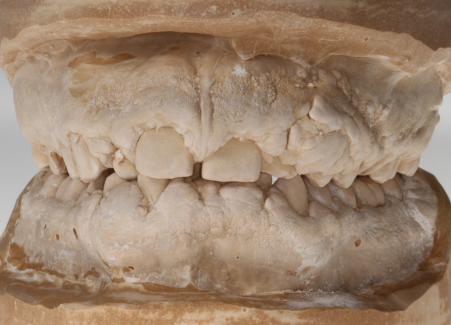

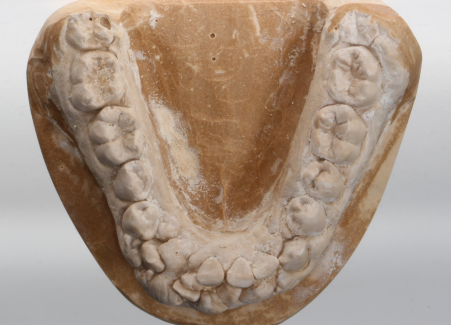

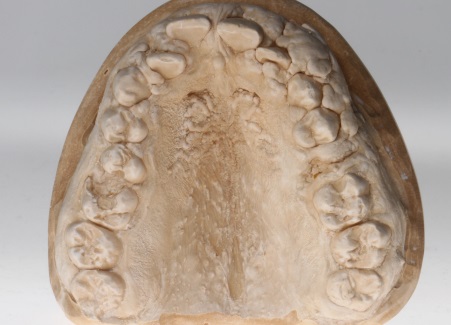

Supplement: Supplementary file 1 [file CCR3-8-159-s001.docx]
