# Supplementary figures and images for: Interdisciplinary assessment and management of a patient with a fibrous gingival enlargement of unknown origin: A case report
Source: Clin Case Rep. 2019 Dec 12;8(1):159–65. doi: 10.1002/ccr3.2605 (PMC6982473; doi:10.1002/ccr3.2605)

**Supplement 2.** Presentation of the patient six months after gingivectomy.

**
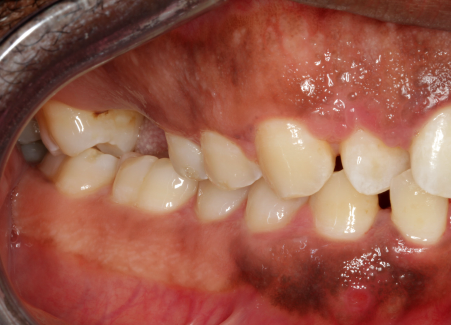

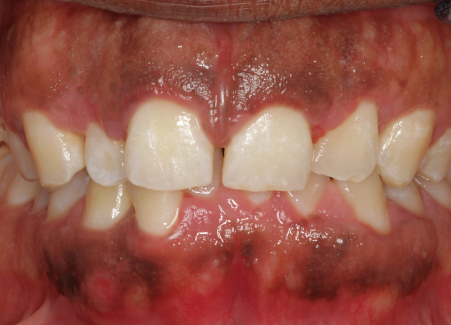

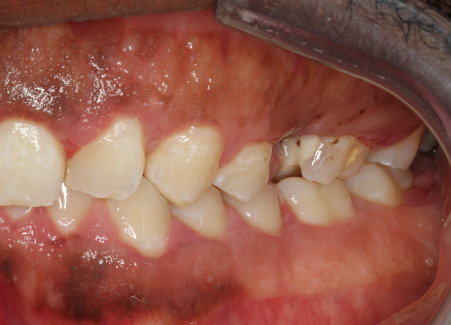
**


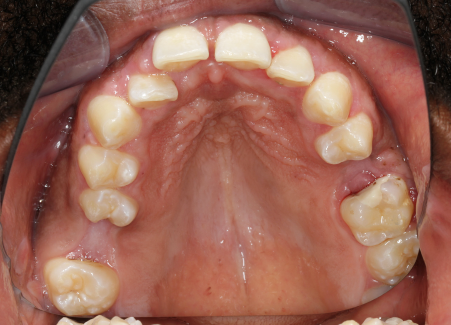

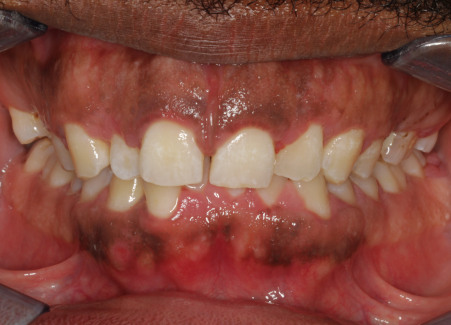

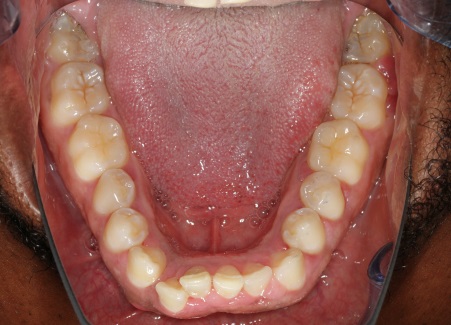

Supplement: Supplementary file 2 [file CCR3-8-159-s002.docx]
